# Supplementary material for: Convergent reductive evolution of cyanobacteria in symbiosis with Dinophysiales dinoflagellates
Source: Sci Rep. 2024 Jun 4;14:12774. doi: 10.1038/s41598-024-63502-0 (PMC11150560; doi:10.1038/s41598-024-63502-0)
Supplement: Supplementary file 1 — Supplementary Information 1. [file 41598_2024_63502_MOESM1_ESM.pdf]

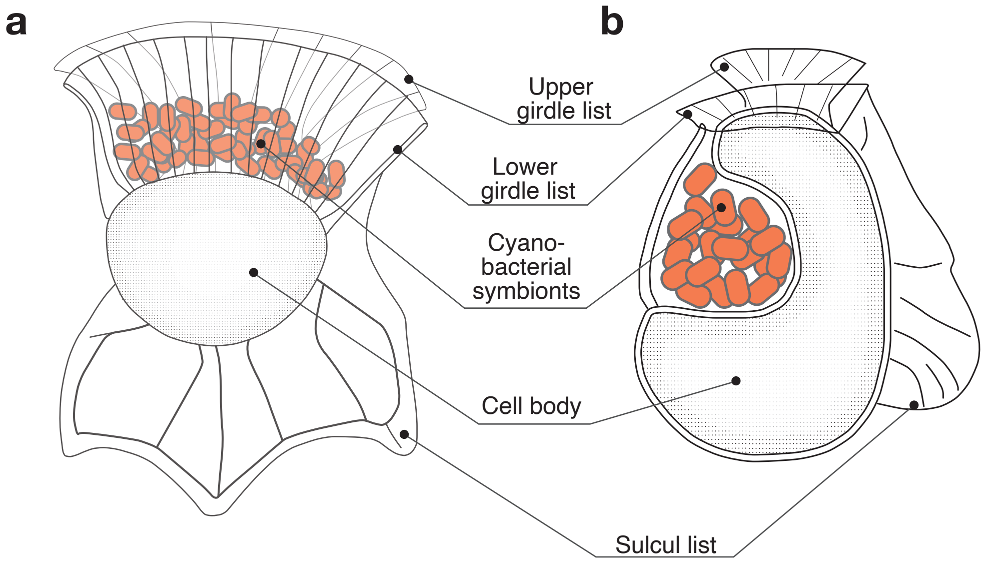

Figure S1. Schematic drawing of the morphology of *Ornithocercus magnificus* **(a)** and *Citharistes regius* **(b)**.
