# Supplementary material for: Convergent reductive evolution of cyanobacteria in symbiosis with Dinophysiales dinoflagellates
Source: Sci Rep. 2024 Jun 4;14:12774. doi: 10.1038/s41598-024-63502-0 (PMC11150560; doi:10.1038/s41598-024-63502-0)
Supplement: Supplementary file 2 — Supplementary Information 2. [file 41598_2024_63502_MOESM2_ESM.pdf]

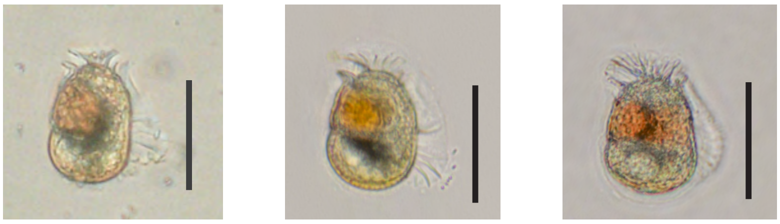

A11 A12 M16

Figure S2. Micrographs of *Citharistes regius* individuals used for whole genome amplification in this study. Scale bars = 40µm.
