# Supplementary material for: Convergent reductive evolution of cyanobacteria in symbiosis with Dinophysiales dinoflagellates
Source: Sci Rep. 2024 Jun 4;14:12774. doi: 10.1038/s41598-024-63502-0 (PMC11150560; doi:10.1038/s41598-024-63502-0)
Supplement: Supplementary file 4 — Supplementary Information 4. [file 41598_2024_63502_MOESM4_ESM.pdf]

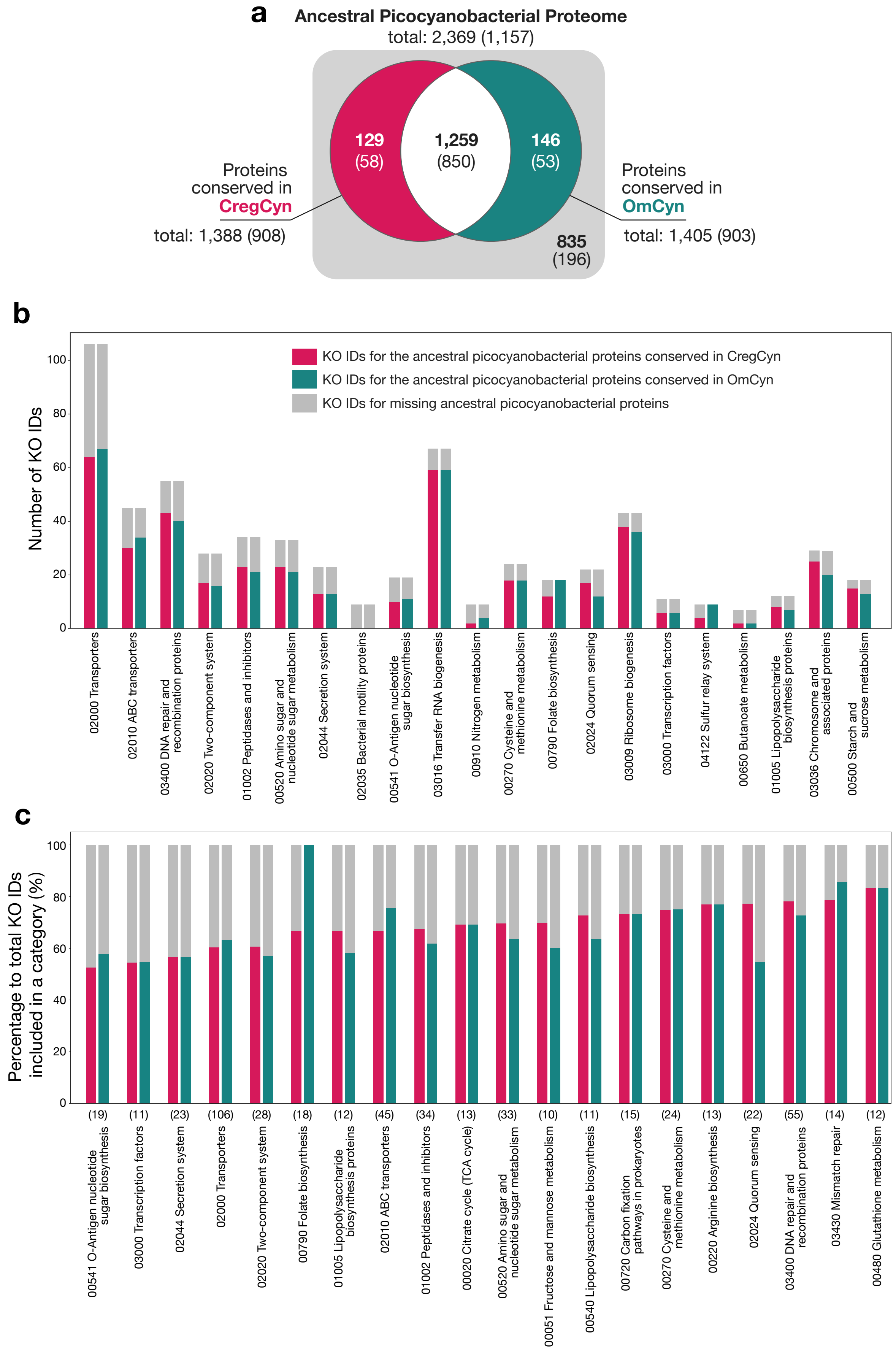

Figure S4. Ancestral picocyanobacterial proteins remaining in the CregCyn and OmCyn genomes. **(a)** Venn diagram of total ancestral picocyanobacterial proteins found in the CregCyn and OmCyn genomes. The numbers in the diagram indicate the number of proteins. Numbers in parentheses indicate the number of proteins that have KEGG Orthology IDs assigned. **(b)** Breakdown by KEGG functional categories. The graph is sorted by the number of KO IDs of proteins lost in CregCyn. Only categories in which five or more KO IDs are missing from the CregCyn or OmCyn genome within the same category are shown. **(c)** Percentage of KO IDs lost or retained per category, sorted by the percentage of lost KO IDs for CregCyn, with only the top 20 shown. The color code is the same as in **b** above; only categories with 10 or more KO IDs in the ancestral picocyanobacterial proteome are shown. Numbers in parentheses below bars represent number of ancestral picocyanobacterial proteome KO IDs for each category.
